# Early Diagnosis of Pulmonary Embolism Related to Clinical Presentation and Vital Signs in the Emergency Department at King Saud Medical City

**DOI:** 10.7759/cureus.27087

**Published:** 2022-07-20

**Authors:** Noman A Khan, Ahad F Alharbi, Ahmed Q Alshehri, Asmaa I Attieh, Habiba H Farouk, Hajr H Alshammri, Haya A Alqahtani, Mai F Alassaf, Malak S Alrejaye, Raneem A Aljthalin, Tassnim S Hafez, Wejdan S Abojalid, Zeyad Zailae, Fatimah M Binsweileh, Ahmed A Alsaleh

**Affiliations:** 1 Emergency, King Saud Medical City, Riyadh, SAU; 2 Emergency Medicine, Al-Maarefa University, Riyadh, SAU; 3 Medicine, King Saud Bin Abdulaziz University for Health Sciences College of Medicine, Riyadh, SAU; 4 Emergency Medicine, Vision College, Jeddah, SAU; 5 Emergency Medicine, AlMaarefa University, Riyadh, SAU

**Keywords:** emergency visits, early predictors, vital signs, clinical presentation, pulmonary embolism

## Abstract

Background: Pulmonary embolism (PE) is a common acute life-threatening cardiovascular disorder. It is the third most common cause of hospital-related death and early detection and management of PE are crucial. The study aimed to evaluate the association between vital signs and laboratory investigations with PE.

Methods: This is a retrospective, hospital records-based, observational study, conducted among patients who were admitted to the emergency department of King Saud Medical City in Riyadh, Saudi Arabia with a suspected diagnosis of PE during the period of March 2021 to March 2022. Data were collected by searching patients’ files and recording demographic data, and information about the clinical presentation, workup, and outcome. Data were entered and analyzed using SPSS version 26 (IBM, Armonk, NY), utilizing Chi-square statistics to test differences between groups, and logistic regression analyses to identify predictors of PE.

Results: The study included 92 patients, with a preponderance of females (70.7%), and those aged 40-60 years (51.1%). Diabetes mellitus (44.6%), and hypertension (30.4%) were the most common comorbidities among others, while shortness of breath (SOB) (83.7%), and chest pain (44.6%) were among the most commonly reported symptoms. A majority of patients had tachycardia (64.1%), while about half had low oxygen saturation (51.5%), and nearly one-third had tachypnea (29.3%), which was more predominant among those not diagnosed with PE. Logistic regression analysis revealed that SOB, respiratory rate, and oxygen saturation were the only significant predictors of PE.

Conclusion: Although being an integral part of the initial assessment in the hospital, measuring the vital signs is not always reflective of the likelihood of PE, and they should not be the only metric relied upon to make decisions about treatment approaches in patients with PE. Physicians should ensure the employment of evidence-based clinical prediction rules and guidelines when diagnosing and managing PE.

## Introduction

Pulmonary embolism (PE) is an acute cardiovascular disorder with high mortality rates despite the advances in detection and management over the last three decades. It is estimated that PE constitutes the third most common cause of hospital-related death and the most common cause of preventable deaths in hospital settings [1,2]. The pathology behind PE is not well established but it is suggested that any impairment in the Virchow's triad (blood stasis, endothelial injury, and hypercoagulable state) plays a role, and the source is usually the deep venous system of the lower limbs as it occurs in about 15%-32% after deep vein thrombosis (DVT) [3,4].

PE can be provoked, in which a risk factor is present, or unprovoked, in which no risk factor has been identified [1]. Risk factors include age, previous history of thromboembolism, malignancy, coagulopathy, prolonged immobilization, surgery, and hormonal replacement therapy [5].

Clinically, PE can range from asymptomatic presentation to presentation with tachypnea, tachycardia, and even fever in cases of small PE. And the more severe form of PE can manifest with additional features of right-sided heart failure, while acute massive PE is characterized by a sudden massive obstruction of the pulmonary bed, hemodynamic instability, and mortality rate of about 20% [6,7].

Due to variable clinical pictures, it may be difficult to diagnose PE and this highlights the importance of considering the possibility of PE. The diagnosis of PE depends on a combination of clinical probability, ECG, imaging techniques, and d-dimer level. Several available guidelines can be utilized to assess the clinical probability including the Wells score, revised Geneva score, and Pulmonary Embolism Rule Out Criteria (PERC) [1]. A meta-analysis was conducted to test the efficacy of combining clinical probability and d-dimer testing in excluding PE. It was found that clinical decision rules and Gestalt’s scoring (the physician's unstructured estimates) can successfully rule out PE if combined with the d-dimer test. The authors recommended the usage of the standardized rule rather than Gestalt’s scoring [8].

Another cohort study was done at the tertiary referral emergency department to emphasize the association between abnormal vital signs and poor clinical outcomes and it showed that abnormal vital signs were predictors of unfavorable outcomes. They concluded that these abnormalities can guide the management plan and communication with the patients and their relatives [9]. While another study failed to formulate any evidence and highlighted the importance of more well-controlled studies in order to answer the research questions [10].

The present study aimed to investigate the association between the routinely monitored vital signs and their abnormalities along with laboratory investigations with the diagnosis of PE among patients attending the tertiary level emergency department of King Saud Medical City in Riyadh, Saudi Arabia.

## Materials and methods

This is a retrospective, hospital records-based, observational study. The study was conducted among patients who were admitted to the emergency department of King Saud medical city in Riyadh, Saudi Arabia with a suspected diagnosis of PE. Criteria for inclusion were patients of both genders, aged 18 years or more, with a clinical suspicion of PE and a Wells score of moderate to high. Patients who were diagnosed with an alternate diagnosis other than PE as the cause of their presentation after undergoing CTPA and patients who died in the course of their admission before reaching a diagnosis were both excluded. Participants were enrolled utilizing a non-probability sampling technique by searching hospital records and including all eligible patients who were admitted during the period of March 2021 till March 2022.

Data were collected by the authors through an author-designed questionnaire. Collected data included socio-demographic characteristics such as age, and gender; information about the clinical presentation of patients including symptoms, vital signs, and comorbid conditions; and information about undergone workup including white blood cell (WBC) count, d-dimer, and troponin levels.

Collected data were entered into Statistical Package for Social Sciences (SPSS) version 26 (IBM, Armonk, NY), after undergoing appropriate coding. Age and numerical vital signs were entered as continuous variables and then classified as per definitions of normal ranges in previous literature [11]. Categorical variables were reported as frequencies (N) and proportions (%), and differences in proportions among groups were tested using Chi-square statistics. Univariate logistic regression analysis was used to identify common predictors associated with the diagnosis of PE, including demographic, clinical, and biochemical parameters. Then, potential predictors were entered into a multivariate logistic regression model utilizing a parsimonious technique including variables with a p-value of < 0.1 from the univariate analysis. Results were expressed as odds ratios (OR) with 95% confidence intervals (CI), and p-values, with a value of ≤ 0.05 as the cut-off for statistical significance.

Ethical approval and consent waivers were obtained from the institutional review board (IRB) at King Saud Medical City (reference number H1RI-17-Apr22-01). In addition, the authors made every effort to ensure that patient data is secure, and that confidentiality is maintained during and after completion of the study, with no inappropriate disclosure of patient information.

## Results

The study included 92 patients who were admitted to the emergency department of King Saud Medical City with a suspected diagnosis of PE. Females constituted the great majority (70.7%) of the study participants. Almost half of the participants (51%) were within the age group of 40-60 years. Diabetes mellitus, hypertension, and DVT were the most commonly reported comorbidities measuring 44.6%, 30.4%, and 21.7%, respectively. All the reported comorbidities of DVT were present among cases with a confirmed diagnosis of PE (Table 1).

**Table 1:** Demographic and coexisting chronic conditions of study subjects Chronic obstructive pulmonary disease (COPD), Systemic lupus erythematosus (SLE)

		Diagnosis of pulmonary embolism						
		Negative		Positive		Total		p (X^2^)
		N	N %	N	N %	N	N %	
Age in years	Less than 40	3	27.3%	12	14.8%	15	16.3%	0.42 (1.7)
	40-60	6	54.5%	41	50.6%	47	51.1%	
	More than 60	2	18.2%	28	34.6%	30	32.6%	
Gender	Male	4	36.4%	23	28.4%	27	29.3%	0.59 (0.3)
	Female	7	63.6%	58	71.6%	65	70.7%	
Comorbidities	Diabetes mellitus	6	54.5%	35	43.2%	41	44.6%	0.48 (0.5)
	Hypertension	6	54.5%	22	27.2%	28	30.4%	0.064 (3.4)
	Malignancy	0	0.0%	8	9.9%	8	8.7%	0.275 (1.2)
	End-stage renal disease	0	0.0%	7	8.6%	7	7.6%	0.31 (1.03)
	Deep vein thrombosis	0	0.0%	20	24.7%	20	21.7%	0.062 (3.5)
	Heart failure	2	18.2%	1	1.2%	3	3.3%	0.003* (8.8)
	Ischemic heart disease	2	18.2%	1	1.2%	3	3.3%	0.003* (8.8)
	COPD	0	0.0%	3	3.7%	3	3.3%	0.52 (0.4)
	SLE	1	9.1%	0	0.0%	1	1.1%	0.006* (7.4)

Regarding the clinical criteria on admission, about 64.1% of the study population had a heart rate of more than 100 BPM. About 66.7% of individuals with a positive diagnosis of PE had a heart rate of more than 100 BPM, while 32.6% of them had a heart rate of 60-100 BPM. Most of the positive cases (75.3%) had a normal respiratory rate while 24.7% reported higher respiratory rate (more than 20). Conversely, more than half (63.6%) of the individuals with a negative diagnosis had higher respiratory rates. The great majority of the participants (88%) had a normal blood pressure, and most of the positive cases (87.7%) had normal blood pressure compared to 90% of the negatives, while only 6.2% of the confirmed PE were found to have high blood pressure. Regarding oxygen saturation, nearly half (51.5%) of the study participants had an oxygen saturation of less than 94%, and about 55.6% of cases with positive results had an oxygen saturation of less than 94%. High temperature (38°C) was evident in 13% of the participants. Of them, 11.1% of positive results had high temperatures compared to 27.3% of the negatives.

In terms of their chief complaint, less than half of positive cases (45.7%) reported chest pain compared to 36.4% of the negative cases. Nearly a third of the study participants (29.3%) reported having a cough and nearly third of the positive cases (27.2%) had a cough compared to negative individuals. Most of our study participants (83.7%) had shortness of breath (SOB). Of them, 88.9% and 45.5% of positive and negative cases respectively reported having a SOB. Fever was evident among 14.8% of the positive cases and 18.2% of negatives, while only 7.4% of the positive cases had leg pain compared to 9.1% of the negatives (Table 2).

**Table 2:** Clinical condition of study subjects on admission

		Diagnosis of pulmonary embolism						
		Negative		Positive		Total		p (X^2^)
		N	N %	N	N %	N	N %	
Heart rate BPM	Less than 60	1	9.1%	2	2.5%	3	3.3%	0.27 (2.6)
	60-100	5	45.5%	25	30.9%	30	32.6%	
	More than 100	5	45.5%	54	66.7%	59	64.1%	
Respiratory rate	Normal (10-20)	4	36.4%	61	75.3%	65	70.7%	0.008* (7.1)
	High (> 20)	7	63.6%	20	24.7%	27	29.3%	
Blood pressure	Low	0	0.0%	5	6.2%	5	5.4%	0.67 (0.8)
	Normal	10	90.9%	71	87.7%	81	88.0%	
	High	1	9.1%	5	6.2%	6	6.5%	
Oxygen saturation	Low (< 94%)	2	18.2%	45	55.6%	47	51.5%	0.02* (5.4)
	Normal (≥ 94%)	9	81.8%	36	44.4%	45	48.9%	
Temperature	Low (< 35.5)	0	0.0%	0	0.0%	0	0.0%	0.135 (2.2)
	Normal (35.5-37.9)	8	72.7%	72	88.9%	80	87.0%	
	High (> 38.0)	3	27.3%	9	11.1%	12	13.0%	
Chief complaint	Chest pain	4	36.4%	37	45.7%	41	44.6%	0.56 (0.34)
	Cough	5	45.5%	22	27.2%	27	29.3%	0.21 (1.6)
	Shortness of breath	5	45.5%	72	88.9%	77	83.7%	< 0.001* (13.4)
	Fever	2	18.2%	12	14.8%	14	15.2%	0.77 (0.08)
	Leg pain	1	9.1%	6	7.4%	7	7.6%	0.84 (0.04)

Investigations results showed a high WBCs count among 38% of participants. Nearly third of positive patients (35.8%) had a higher count compared to 54.5% of negative patients. About 71% have had a higher D-dimer distributed as 70.5% and 75% of positive and negative cases respectively. Similarly, more than half (52.9%) had a positive troponin result distributed as 44.8% and 100% of positive and negative cases respectively, while lactate levels were high in 63%, constituting 64.2% of patients diagnosed with PE.

Almost all cases 98.9% were admitted to the hospital including all of the positive cases while only one individual with a negative result has been discharged. About 93.4% of the study population were admitted to the general ward including 93.8% of the positive cases and 90% of the negative cases, while 8.6% of the positive cases were admitted to the ICU compared to 10% of those with negative results. About 15.2% died in the inpatient settings, which constituted 17.3% of the positive cases. No death has been reported among the negative cases (Table 3).

**Table 3:** Investigation results and outcome of presentation of study subjects

		Diagnosis of pulmonary embolism						
		Negative		Positive		Total		p (X^2^)
		N	N %	N	N %	N	N %	
WBCs	Low	0	0.0%	0	0.0%	0	0.0%	0.23 (1.4)
	Normal	5	45.5%	52	64.2%	57	62.0%	
	High	6	54.5%	29	35.8%	35	38.0%	
D-dimer	Low	0	0.0%	0	0.0%	0	0.0%	0.79 (0.07)
	Normal	2	25.0%	18	29.5%	20	29.0%	
	High	6	75.0%	43	70.5%	49	71.0%	
Troponin	Low	0	0.0%	1	3.4%	1	2.9%	0.074 (5.2)
	Normal	0	0.0%	15	51.7%	15	44.1%	
	High	5	100.0%	13	44.8%	18	52.9%	
Lactate	Normal	5	45.5%	29	35.8%	34	37%	0.534 (0.387)
	High	6	54.5%	52	64.2%	58	63%	
Deposition	Discharge	1	9.1%	0	0.0%	1	1.1%	0.006* (7.4)
	Admission	10	90.9%	81	100.0%	91	98.9%	
Admission to the general ward	Yes	9	90.0%	76	93.8%	85	93.4%	0.645 (0.2)
	No	1	10.0%	5	6.2%	6	6.6%	
Admission to the ICU	Yes	1	10.0%	7	8.6%	8	8.8%	0.89 (0.02)
	No	9	90.0%	74	91.4%	83	91.2%	
Inpatient death	Yes	0	0.0%	14	17.3%	14	15.2%	0.134 (2.2)
	No	11	100.0%	67	82.7%	78	84.8%	

When analyzing predictors of PE, univariate logistic regression analysis revealed that statistically significant clinical predictors included presentation with SOB (OR = 9.6, 95% CI: 2.43 - 37.9; p = 0.001), respiratory rate > 20 (OR = 5.337, 95% CI: 1.41 - 20.1; p = 0.013), and oxygen saturation of ≥ 94% (OR = 0.178, 95% CI: 0.036 - 0.875; p = 0.034). However, on multivariate analysis, respiratory rate was omitted from the model due to multicollinearity, while SOB (aOR = 12.99, 95% CI: 2.76 - 61.1; p = 0.001) was the only remaining significant predictor (Table 4).

**Table 4:** Demographic, clinical, and biochemical predictors of pulmonary embolism according to univariate and multivariate regression analyses

		Univariate analysis			Multivariate analysis		
		OR	95% CI	p	OR	95% CI	p
Age (years)	< 40	1					
	40 - 60	1.7	0.37 – 3.9	0.492			
	> 60	3.5	0.52 – 23.7	0.199			
Gender	Male	1					
	Female	1.4	0.39 – 5.4	0.588			
Presenting manifestations	Chest pain	1.47	0.399 – 5.4	0.561			
	Cough	0.447	0.124 – 1.6	0.22			
	SOB	9.6	2.43 – 37.9	0.001*	6.84	1.6 – 28.6	0.008*
	Fever	0.783	0.15 – 4.08	0.771			
	Leg pain	0.8	0.087 – 7.3	0.844			
Heart rate	60 - 100	1					
	< 60	2.5	0.188 – 33.2	0.487			
	> 100	5.4	0.41 – 70.5	0.198			
Respiratory rate	Normal (10-20)	1					
	High (> 20)	5.337	1.41 – 20.1	0.013*	-	-	-
Oxygen saturation	Low (< 94%)	1					
	Normal (≥ 94%)	0.178	0.036 – 0.875	0.034*	0.279	0.052 – 1.49	0.136
Temperature	Normal (35.5-37.9)	1					
	High (> 38.0)	0.3	0.075 – 1.49	0.15			
WBCs	Normal	1					
	High	0.465	0.13 – 1.66	0.237			
d-Dimer	Normal	1					
	High	0.796	0.147 – 1.3	0.792			
Lactate	Normal	1					
	High	1.494	0.419 – 5.3	0.536			

## Discussion

PE is a major health problem encountered in the emergency room (ER) setting. It is the third leading cause of cardiovascular-related death after myocardial infarction and stroke [12]. The assessment of patients with this disorder passes through many processes that utilize new information and updates on the existing ones in order to make a diagnosis, and most clinicians will initially rely on the vital signs as a part of this process [13,14].

In line with previous literature, our study report that SOB is the most common presenting symptom of PE. Statistical analysis showed that 83.7% of the study population reported having SOB (p<0.001). A similar finding is in line with another two studies where 64% and more than 85% have been reported [1,15].

Although only 55.6% of positive cases had an oxygen saturation of less than 94%, univariate logistic regression revealed that oxygen saturation ≥ 94% is a significant negative predictor of PE, while it was not significant in the multivariate model, in which SOB was still a significant predictor. In a previous study, Kline et al. reported that oxygen saturation was the most important predictor of death among patients with PE, and concluded that saturation of equal to or more than 95 measured by the pulse oximetry was associated with a reduced possibility of hospital complications from PE [13].

A considerable number of positive diagnoses of PE had a normal measurement of vital signs despite their confirmed diagnosis of PE. This finding highlights an important fact that clinicians should not only rely on these measurements and forget the need for objective testing. The issue of normalization of vital signs among patients with PE was studied by Kline et al, and although the initial reading of oxygen saturation had higher predictability of PE, other vital signs measurements were normal in many patients [13].

D-dimer is the final product of fibrin degradation, its level rises in response to the presence of a blood clot in the circulatory system. Hence, a normal result of d-dimer should technically rule out the possibility of DVT or PE. In our study, 70.5% of the confirmed cases of PE had an elevated d-dimer and the remaining third of the cases were found to have normal levels of d-dimer. This was a higher rate of false negatives compared to what is reported in a retrospective study that investigated the use of d-dimer for detecting PE in the emergency department. The study found that d-dimer testing was associated with 9 false negatives out of 1,270 patients on which imaging was done and confirmed the diagnosis of PE. However, the d-dimer test showed a sensitivity of 95.7% and a specificity of 40% [1,16]. Another study that aimed to estimate the prevalence of d-dimer utilization in the ER revealed that there was a lack of adherence to the guidelines in terms of d-dimer testing before imaging with CT pulmonary angiography [17].

About 45% of the positive cases of PE were found to have higher levels of troponin. It is believed that measuring the level of troponin (originally a cardiac enzyme that is used generally to detect any myocardial insult) is a useful reflection in predicting the severity of PE. It was also considered to be associated with higher mortality. Likewise, a meta-analysis study performed to investigate the risk of elevated troponin among patients with PE pointed out that the risk is increasing to five-fold with any increase in the level of troponin [1,18]. Another meta-analysis showed the same findings with a 4-8 folds increase in the risk [19]. Moreover, the overall in-patient hospital mortality was 15.2% in our study, a significantly higher rate compared to the 5.6% reported in Jan et al.'s study [1].

The research does have certain limitations. First, because database analysis was retrospective, it was challenging to gather information and pinpoint the exact cause of PE, and stratify patients based on severity. Additionally, the data collection was done using a manual method of collection and was not computerized, which may be more prone to error. As a result, some information was missing. Moreover, triage vital signs were taken into account, with some patients having a prolonged stay in the emergency department, therefore it would not be ideal to employ triage vital signs in these cases. Finally, the sampling technique was non-probability sampling thus the population may not be adequately represented.

## Conclusions

Although being an integral part of the initial assessment in the hospital, measuring the vital signs is not always reflective of the likelihood of PE, and they should not be the only metric relied upon to make decisions about treatment approaches in patients with PE. Physicians should ensure the employment of evidence-based clinical prediction rules and guidelines when diagnosing and managing PE.
